# Influence of the Human Lipidome on Epicardial Fat Volume in Mexican American Individuals

**DOI:** 10.3389/fcvm.2022.889985

**Published:** 2022-06-06

**Authors:** Ana Cristina Leandro, Laura F. Michael, Marcio Almeida, Mikko Kuokkanen, Kevin Huynh, Corey Giles, Thy Duong, Vincent P. Diego, Ravindranath Duggirala, Geoffrey D. Clarke, John Blangero, Peter J. Meikle, Joanne E. Curran

**Affiliations:** ^1^Department of Human Genetics and South Texas Diabetes and Obesity Institute, University of Texas Rio Grande Valley School of Medicine, Brownsville, TX, United States; ^2^Eli Lilly and Company, Indianapolis, IN, United States; ^3^Metabolomics Laboratory, Baker Heart and Diabetes Institute, Melbourne, VIC, Australia; ^4^Baker Department of Cardiometabolic Health, University of Melbourne, Parkville, VIC, Australia; ^5^Department of Radiology and Research Imaging Institute, University of Texas Health Science Center, San Antonio, TX, United States

**Keywords:** cardiovascular disease, lipidomic profiles, epicardial fat volume, Mexican Americans, CMRI

## Abstract

**Introduction:**

Cardiovascular disease (CVD) is the leading cause of mortality worldwide and is the leading cause of death in the US. Lipid dysregulation is a well-known precursor to metabolic diseases, including CVD. There is a growing body of literature that suggests MRI-derived epicardial fat volume, or epicardial adipose tissue (EAT) volume, is linked to the development of coronary artery disease. Interestingly, epicardial fat is also actively involved in lipid and energy homeostasis, with epicardial adipose tissue having a greater capacity for release and uptake of free fatty acids. However, there is a scarcity of knowledge on the influence of plasma lipids on EAT volume.

**Aim:**

The focus of this study is on the identification of novel lipidomic species associated with CMRI-derived measures of epicardial fat in Mexican American individuals.

**Methods:**

We performed lipidomic profiling on 200 Mexican American individuals. High-throughput mass spectrometry enabled rapid capture of precise lipidomic profiles, providing measures of 799 unique species from circulating plasma samples. Because of our extended pedigree design, we utilized a standard quantitative genetic linear mixed model analysis to determine whether lipids were correlated with EAT by formally testing for association between each lipid species and the CMRI epicardial fat phenotype.

**Results:**

After correction for multiple testing using the FDR approach, we identified 135 lipid species showing significant association with epicardial fat. Of those, 131 lipid species were positively correlated with EAT, where increased circulating lipid levels were correlated with increased epicardial fat. Interestingly, the top 10 lipid species associated with an increased epicardial fat volume were from the deoxyceramide (Cer(m)) and triacylglycerol (TG) families. Deoxyceramides are atypical and neurotoxic sphingolipids. Triacylglycerols are an abundant lipid class and comprise the bulk of storage fat in tissues. Pathologically elevated TG and Cer(m) levels are related to CVD risk and, in our study, to EAT volume.

**Conclusion:**

Our results indicate that specific lipid abnormalities such as enriched saturated triacylglycerols and the presence of toxic ceramides Cer(m) in plasma of our individuals could precede CVD with increased EAT volume.

## Introduction

Cardiovascular disease (CVD) is the leading cause of mortality worldwide and remains the leading cause of death in the US (1, 2). CVD encompasses a broad range of disorders of the cardiovascular system, including coronary heart disease, cerebrovascular disease, and peripheral artery disease. Underlying them all is atherosclerosis (ATS)—a complex disorder in which a host of different intrinsic and extrinsic processes and factors contribute to the development of lesions that eventually compromise normal vascular function (3). ATS is an inflammatory disease of the arteries associated with lipid and other metabolic alterations (4). Lipid dysregulation is a well-known precursor to metabolic diseases (MeD), including CVD. Risk factors for ATS and CVD, including age, sex, lipid levels, smoking and blood pressure, are incorporated in risk algorithms that are used to predict an individual's absolute risk for CVD in the general population (5). Although these risk factors are useful to predict disease risk in populations, their accuracy in predicting cardiovascular risk in individuals varies considerably across populations (6). Mexican Americans have a high prevalence of cardiovascular morbidity and mortality (5, 7). The high risk for CVD in this ethnic group is partly explained by a high propensity to MeD (5).

Evidence from epidemiological and lipidomic studies has shown that specific lipoproteins and their constituent lipids are important factors in the development of metabolic related disorders (8–10), including CVD (11–13). The classical lipid traits (such as HDL-C, LDL-C, and triglycerides), most commonly examined in disease risk, are complex molecules comprised of multiple lipid and protein components (14). The lipidome is the total complement of these lipid species. Even though these lipid measures are influenced by both environmental and genetic factors, genetic variation is a significant determinant with traditional plasma measures having heritability of more than 50% (15, 16). Although CVD risk is heritable and there have been a number of successes localizing QTLs that influence disease risk, the genetic basis of this risk is still relatively unknown. Quantitative endophenotypes that are related to disease liability can offer more power for gene localization/identification than dichotomous disease status and thus serve as valuable phenotypes for disease gene identification. Such endophenotypes, whose alterations precede disease, may also be useful for early risk assessment. Measures of the human lipidome represents a wealth of phenotypes that may be better predictors of disease than the traditional clinical measures (17). The biologically simpler nature of these individual species suggests that they may reside closer to the causal action of genes, making them valuable endophenotypes (18). Lipidomic studies are helping to decipher the complex interactions between lipid metabolism and MeD, and identifying pathways representing potential therapeutic targets not seen with studies of traditional lipids (19).

Intermediate phenotypes such as those derived by CMRI and lipid profiling lie closer to the genomic level and the causal action of genes. Organ-specific adiposity has renewed growing interest in that and contributes to the pathophysiology of cardiometabolic diseases (20, 21). However, the molecular changes in adipose tissue that promote these disorders are not completely understood and, in particular, the specific role of cardiac adiposity. Pericardial adipose tissue is ectopic fat in the mediastinum, which is associated with poor metabolic and cardiac health, especially in with type II diabetes mellitus (T2DM) patients (22, 23). Pericardial adipose tissue is comprised of two compartments. Epicardial adipose tissue (EAT) is defined as the visceral fat deposited between the epicardium and the pericardium. Paracardial adipose tissue (PAT) is the fat deposited on the external surface of the pericardium within the mediastinum (24). Growing evidence indicates that epicardial adipose tissue (EAT) volume is positively associated with coronary artery disease (23). The increase of EAT has been associated with different cardiovascular risk factors (25) such as type 2 diabetes mellitus, hypertension, and obesity, in isolation or as part of the MeD (26–28). Growing evidence indicates that EAT volume is positively associated with coronary artery disease (29), but the mechanisms involved in this relationship remain largely unknown. EAT is the visceral fat deposit over the heart, located between the myocardium and the visceral pericardium (21, 30). EAT is more than a simple fat storage depot. Indeed, it is now widely recognized to be an extremely active endocrine organ, producing cytokines, adipokines, and chemokines that can be either protective or harmful depending on the local microenvironment (31, 32). These functions highlight the complex cellularity and crosstalk between EAT and neighboring structures (21). EAT metabolism is uniquely regulated due to the high basal rates of fatty acid uptake, insulin-induced lipogenesis, and high fatty acid breakdown (33, 34). EAT may act as a local energy store for cardiac muscle and has a protective role against elevated levels of free fatty acid (FFA) in the coronary circulation (35). Additionally, EAT could influence coronary atherogenesis and myocardial function because there is no fibrous fascial layer to impede the diffusion of FFA and adipokines between it and the underlying vessel wall as well as the myocardium (34, 36). EAT is also actively involved in lipid and energy homeostasis, with EAT having a greater capacity for release and uptake of FFA (36–38). FFA synthesis and rates of incorporation and breakdown are significantly higher in EAT than in other fat deposits. However, the amount and type of fatty acids stored could also play a role in the onset and development of atherosclerosis (39). These metabolites serve as direct signatures of biochemical activity, being, therefore, easier to correlate with quantitative plasma lipid phenotypes. In this context, lipidomics has become a powerful approach to mechanistically investigate how biochemistry relates to clinical phenotype (40, 41). We concentrate on EAT because it is a known adiposity-related risk factor for CVD, directly related to heart function, but has been comparatively less studied than more easily measured fat measures. However, there is a scarcity of information on the interaction of plasma lipids and CMRI EAT measures. Therefore, this study identifies plasma lipidomic species associated with CMRI-derived measures of epicardial fat, as a measure of CVD risk, in a cohort of Mexican American individuals.

## Materials and Methods

### Study Participants

The SAFHS began in 1991 and was initially designed to investigate the genetics of CVD in Mexican Americans. The SAFHS enrolled large, extended Mexican Americans families residing in San Antonio, TX, and ascertainment occurred by way of the random selection of an adult Mexican American proband, without regard to disease. The enrollment procedures, inclusion and exclusion criteria, and phenotypic assessments of the study participants have been described in detail previously (7, 42). This is an ongoing investigation, which has had four phases of data collection over a 25-year period. The data and samples used in this study were collected during the first phase of data collection, occurring between 1992 and 1996. Blood samples were collected from participants on their visit day using standard procedures, and participants were fasted for 12 h. For plasma, blood was collected in a K2-EDTA vacutainer, and the plasma layer removed after centrifugation. Plasma was stored in 50 μL aliquots at −80°C and has not been thawed post-collection. In the current study, 200 individuals that have both lipidome profile measures and CMRI-derived epicardial fat data were included. Of these 200 SAFHS participants, 63% were female, and the sample was aged between 18 and 83 (46.85 ± 16.23). Informed consent was obtained from all participants before collection of samples. The study conformed to the Declaration of Helsinki, and the Institutional Review Boards of the University of Texas Health Sciences Center at San Antonio and the University of Texas Rio Grande Valley approved this study.

### Epicardial Fat Tissue Measurement by Cardiac Magnetic Resonance Imaging

Cardiac imaging was performed on a 3.0 Tesla MR scanner (TIM Trio; Siemens, Erlangen, Germany) with a 6-channel anterior matrix torso coil combined with posterior spine-coil elements producing an aggregate of 12 data channels. For MRI of the abdominal aorta, an additional 6-channel anterior matrix receiver coil ensures full coverage. All subject assessment, blood collection, and imaging were performed at the Research Imaging Institute at UTHSCSA. Before each session, a standard quality control phantom was imaged. Cardiac morphology and function were determined after initial anatomical localizer images. High temporal resolution cine MR imaging with prospective gating was performed, using a balanced, steady-state free precession sequence (TR/TE = 2.44/1.22 ms, 25–30 cardiac phases, matrix 224 × 288, FOV 336 × 430 mm^2^, pixel resolution 1.5 × 1.5 mm^2^). Two 3-slice, cine, long-axis data sets in a left anterior oblique and a four-chamber view were acquired. A stack of contiguous short-axis slices, extending from the apex of the LV through the atria, are then acquired with repetitive end expiration breath-holds. Heart rate and EKG were monitored during the CMRI. Pericardial fat scans were obtained in diastole using a short-axis, single-shot steady-state free precession (IR-SFFP) sequence with an inversion-recovery pre-pulse (TR/TE = 2.4/1.1 ms, slice = 8.5 mm, 1.3 × 1.3 mm pixel, flip angle = 50°) that covered the complete left ventricle by long-axis stacks in a single breath-hold. The inversion time (TI) was adjusted between 180 and 280 ms to obtain optimal enhancement of the epicardial fat. The cvi42 image analysis package (Circle Cardiovascular Imaging Inc., Calgary, AB) was used for assessment of pericardial fat. Using long axis, 4-chamber images, thickness of pericardial fat was measured at the cardiac mid-ventricular level laterally. For analyses of pericardial fat, manual ROI contours were drawn to define the EAT area by the region included inside contours of the epicardium and pericardium. Summation of the pixels included in the contours was recorded and multiplied by the per-pixel dimensions to obtain an area measurement. For epicardial fat volume determination, the area subtended by the manual tracings is determined in each slice and multiplied by the slice thickness and then summed to yield the fat volume. Visceral fat was not examined in this study, it is measured in the abdomen and our imaging protocol did not capture the abdominal region.

### Lipidomic Profiling

Lipidomics was performed as described previously (43) with modifications. Analysis of plasma extracts was performed on an Agilent 1290 series HPLC system coupled to an Agilent 6495C triple quadrupole mass spectrometer. Liquid chromatography was performed on a dual column system with ZORBAX eclipse plus C18 columns (2.1 × 100 × 1.8 mm, Agilent) heated to 45°C. Mass spectrometry analysis was performed in both positive and negative ion mode with dynamic scheduled multiple reaction monitoring (MRM). The running solvent consisted of solvent A: 50% H2O/30% acetonitrile/20% isopropanol (v/v/v) containing 10 mM ammonium formate and 5 μM medronic acid, and solvent B: 1% H2O/9% acetonitrile/90% isopropanol (v/v/v) containing 10 mM ammonium formate. Gradient conditions are presented in Supplementary Table S1, with alternating columns for sample analysis (Pump A) and equilibration (Pump B). The following mass spectrometer conditions were used: gas temperature 150°C, gas flow rate 17 L/min, nebulizer 20 psi, sheath gas temperature 200°C, capillary voltage 3,500 V and sheath gas flow 10 L/min. Isolation widths for Q1 and Q3 were set to “unit” resolution (0.7 amu). Plasma quality control (PQC) samples consisting of a pooled set of plasma samples taken from six healthy individuals and extracted alongside the study samples were incorporated into the analysis at 1 PQC per 20 plasma samples. NIST 1950 SRM sample (Sigma) was included as a reference plasma sample, at a rate of 1 per 40 samples. Relative quantification of lipid species was determined by comparison to the relevant internal standard. Lipid class total concentrations were calculated as the sum of individual lipid species concentrations, except in the case of two lipid classes, triacylglycerol (TG) and alkyldiacylglycerol, TG(O), where we measured both neutral loss (NL) and single ion monitoring (SIM) transitions. We used the more quantitatively accurate but less structurally resolved (SIM) species concentrations for summation purposes when examining lipid totals, while their (NL) counterparts were used for associations.

### Statistical Analysis

Initially, we estimated epicardial fat volume content heritability using a quantitative genetic variance component decomposition approach as implemented on SOLAR (version 8.1.1) (44). We used a linear mixed model $\Omega =2\phi \sigma_{G}^{2}+I\sigma_{e}^{2}$ where, Ω is the total phenotypic covariance matrix of a trait, ϕ is the matrix of kinship coefficients, *I* is the identity matrix, $\sigma_{G}^{2}$ is additive genetic variance, and $\sigma_{e}^{2}\;$is residual environmental variance. All the models were adjusted for the following covariates: age, age^2^, sex, age × sex, and age^2^ × sex. We do not use medication as a covariate. This cohort is a traditionally medically underserved population and very few of them are taking lipid lowering or other relevant medications (we collect medications at all subject visits). When we correct for medications, the observed results are not qualitatively changed. Heritability was defined as the proportion of phenotypic variance explained by an additive genetic model. We tested the association of each lipid species and lipid classes with epicardial fat volume by means of LRT (Likelihood Ratio Test) comparing two models with and without the presence of a lipid class/species while allowing for residual non-independence due to underlying genetic factors which induce correlations between related individuals. Statistical significance of a given lipid association with epicardial fat was tested by constraining the regression coefficient to 0 and comparing the log-likelihoods of the constrained and unconstrained regression models to yield an LRT test that is distributed as χ^2^ variate with one degree of freedom. The significant lipid classes and species were defined using a False Discovery Rate (FDR) smaller than 0.05 calculated using the empirical *p*-value distribution.

## Results

For this study, we had a cohort of 200 Mexican American individuals with lipidomic profiles and CMRI-derived epicardial fat volume measures. Baseline demographic and clinical status of these 200 participants is shown in Table 1. The mean age of the participants was 46.85 ± 16.23 years old, 74 (37%) were male, 73 (36.5%) had hypertension, and 43 (21.5%) had type 2 diabetes (T2D). Mean body mass index (BMI) was 32.158 ± 6.40 kg/m^2^. There were no differences in BMI, prevalence of hypertension or T2D between men and women, however the prevalence of T2D in women was 2.5 times higher than in men.

**Table 1:** Characteristics of the study population.

**Parameters**	**Average**	**Minimum**	**Maximum**
**Sex**			
Male (*n*-%)	74 (37)	–	–
Female (*n*-%)	126 (63)	–	–
Age (years old)	46.85 ± 16.23	18	83
Male	45.18 ± 17.10	19	77
Female	47.83 ± 15.62	18	83
Education (years)	12.39 ± 2.94	1	22
Height (cm)	161.96 ± 9.54	127.00	186.50
Weight (Kg)	84.44 ± 18.76	39.92	163.29
BMI	32.158 ± 6.40	17.51	51.25
Waist (cm)	101.56 ± 14.82	63.00	149.00
Systolic BP-Avg (mmHg)	125.24 ± 16.82	96.00	191.67
Diastolic BP-Avg (mmHg)	76.24 ± 11.58	40.33	142.00
Diabetes mellitus (*n*-%)	43 (21.5%)	–	–
Hypertension (*n*-%)	73 (36.5%)	–	–

*n, number of participants; %, percentage; cm, centimeters; Kg, Kilograms; mmHg, millimeters of mercury*.

**Figure 1:**
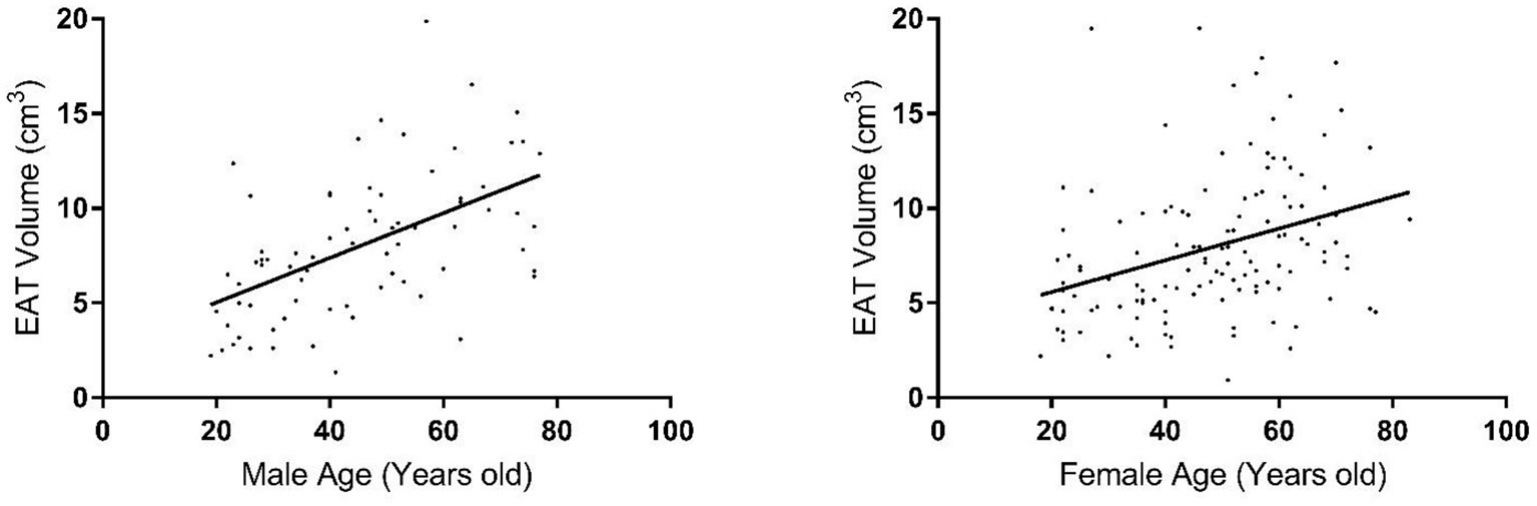
Distribution of epicardial fat volume in males **(left plot)** and females **(right plot)** by age group.

EAT volume was evaluated with good reproducibility in our study. Average EAT volume was 7.95 ± 3.81 cm^3^ in all individuals. EAT volume was not significantly different in hypertensive vs. normotensive subjects (8.85 ± 3.73 cm^3^ vs. 7.45 ± 3.74 cm^3^, *p* = 0.6699); nor was it significantly different between subjects with and without T2D (9.32 ± 4.0 cm^3^ vs. 7.6 ± 3.65 cm^3^, *p* = 0.2812). In this study, the mean EAT volume was 7.95 ± 3.81 cm^3^, ranging from 0.94 to 19.88 cm^3^. The age-adjusted difference in EAT volume between males and females was trending toward significance (8.0 ± 3.74 cm^3^ vs. 7.92 ± 3.84 cm^3^, *p* = 0.0561). After analyzing the sample by increasing age, we observed a significant increase in EAT volume with increasing age, in both males and females (*p* < 2.5 × 10^−6^) (Figure 1). There was no evidence for any interactions of sex and age. Additionally, there was no significant evidence for a non-linear relationship with age.

Quantitative genetic analysis revealed that EAT is under substantial genetic control in this population. The estimated heritability was 0.86 ± 0.16, 95% CI = (0.55, 1.0), *p* = 1.8 × 10^−6^. To our knowledge, this is the first study to evaluate the relative importance of additive genetic factors for this phenotype in humans.

To optimize the number of statistical tests being performed, we first conducted our analyses at the level of lipid class. We analyzed the association of 48 lipid classes listed in Table 2 with epicardial fat volume. Using multivariable regression models performed in SOLAR and controlling the false discovery rate (FDR) using the Benjamini-Hochberg method (45–47), we found that nine lipid classes were statistically associated with EAT volume (Table 3). The classes were triacylglycerol (TG), hydroxylated acylcarnitine (AC-OH), deoxyceramide (Cer(m)), alkyldiacylglycerol (TG(O)), ubiquinone, diacylglyceride (DG), dihydroceramide (dhCer), phosphatidylinositol (PI) and phosphatidylglycerol (PG), respectively by lowest to highest FDR < 0.05. It is worth noting that several of the classes associated with EAT volume were previously implicated in the pathogenesis of T2D, insulin resistance and consecutively in CVD risk.

**Table 2:** Lipid classes assessed by targeted lipidomics: concentration (pmol/mL), range of concentration (maximum and minimum, pmol/mL) and associations with EAT volume.

**Lipid class name**	**Class abbreviation**	**Concentration**	**Minimum**	**Maximum**	**No. of species**	No. of species^*^
Acylcarnitine	AC	780.16	277.75	1,897.03	28	4
Alkenylphosphatidylcholine (P)	PC(P)	60,236.05	25,391.79	128,139.69	30	0
Alkenylphosphatidylethanolamine (P)	PE(P)	63,098.10	22,049.00	157,529.53	53	0
Alkyldiacylglycerol	TG(O)	2,225.81	526.33	17,015.81	20	12
Alkylmonoacylglycerol	DG(O)	1,931.96	442.86	5,054.66	2	0
Alkylphosphatidylcholine	PC(O)	71,020.49	35,800.19	125,404.02	34	0
Alkylphosphatidylethanolamine	PE(O)	3,595.49	1,510.84	7,742.28	14	0
Bile acid	BA	835.86	115.07	6,533.81	2	0
Ceramide	Cer(d)	18,222.47	5,289.50	35,327.51	47	8
Ceramide-1-phosphate	C1P	5.95	0.96	17.30	1	0
Cholesteryl ester	CE	3,481,161.11	1,801,143.14	6,008,721.67	27	2
Dehydrocholesterol ester	DE	18,330.58	7,819.13	61,318.78	6	1
Dehydrodemosterol ester	deDE	360.13	23.77	5,225.42	2	0
Deoxyceramide	Cer(m)	1,337.83	258.05	7,013.12	11	8
Diacylglycerol	DG	113,181.88	37,769.76	432,782.54	26	14
Dihexosylceramide	Hex2Cer	4,007.89	1,549.11	7,162.76	10	0
Dihydroceramide	dhCer	375.41	149.99	777.79	6	2
Dimethyl-cholesteryl ester	dimethyl-CE	24,635.70	7,573.76	71,080.92	4	0
Free cholesterol	COH	747,839.09	428,202.30	1,178,895.10	1	0
Free fatty acid	FFA	395,032.85	205,376.16	683,429.45	17	0
GD1 ganglioside	GD1	7.36	1.84	14.66	1	0
GM1 ganglioside	GM1	19.03	0.61	41.33	1	0
GM3 ganglioside	GM3	3,121.22	1,384.39	5,629.32	7	0
Hydroxylated acylcarnitine	AC-OH	23.52	9.22	46.06	8	5
Lysoalkenylphosphatidylcholine (P)	LPC(P)	772.16	198.42	1,508.36	6	0
Lysoalkenylphosphatidylethanolamine (P)	LPE(P)	310.79	102.04	745.05	4	0
Lysoalkylphosphatidylcholine (LAF)	LPC(O)	3,094.26	1,333.28	4,890.32	10	0
Lysophosphatidylcholine	LPC	219,759.08	115,804.17	370,825.40	61	3
Lysophosphatidylethanolamine	LPE	6,563.27	3,124.24	12,965.85	14	0
Lysophosphatidylinositol	LPI	1,519.93	844.58	2,776.85	7	2
Methyl-cholesteryl ester	methyl-CE	37,950.13	10,953.57	129,335.37	5	0
Methyl-dehydrocholesteryl ester	methyl-DE	2,929.83	622.81	9,579.89	2	0
Monohexosylceramide	HexCer	3,212.20	911.79	6,392.99	14	0
Oxidized lipids	OxSpecies	6,579.85	2,681.95	13,155.84	6	1
Phosphatidic acid	PA	76.43	40.32	164.48	5	0
Phosphatidylcholine	PC	1,737,177.86	1,048,144.69	2,398,255.66	90	4
Phosphatidylethanolamine	PE	40,484.40	15,353.48	95,420.85	37	5
Phosphatidylglycerol	PG	475.99	134.61	1,170.81	3	1
Phosphatidylinositol	PI	215,820.31	60,717.32	370,648.86	27	9
Phosphatidylinositol monophosphate	PIP1	628.26	85.24	1,380.75	1	0
Phosphatidylserine	PS	6,981.00	354.86	23,312.22	7	0
Sphingomyelin	SM	342,792.34	190,455.47	557,621.10	47	2
Sphingosine	Sph	494.68	214.47	1,108.70	1	0
Sphingosine-1-phosphate	S1P	2,072.79	1,120.77	4,252.17	4	0
Sulfatide	SHexCer	565.90	269.16	1,082.07	6	0
Triacylglycerol (NL)	TG	1,742,464.14	450,098.99	7,784,888.83	77	51
Trihexosylcermide	Hex3Cer	1,580.81	739.32	2,721.76	6	0
Ubiquinone	Ubiquinone	2,069.76	508.35	4,924.12	1	1
**Total**					**799**	**135**

*Numeric values are given as average and range. No., number; P, Plasmogen; LAF, lysoplatelet activating factor*.

* *No. with p < 0.05 designates the number of lipids significantly associated with EAT volume measurement at the 0.05 level of false discovery rate (FDR) under adjustment for age, sex and their interactions*.

**Table 3:** Species and classes of lipid with the lowest false discovery rate *p-*values in our study.

**Lipid classes**				
**Lipid classes name**	**Lipid abbreviation**	***p*** **-value**	**Beta (SDU)**	**FDR**
Triacylglycerol	TG	3.23E-04	0.3062	6.97E-03
Alkyldiacylglycerol	TG(O)	1.07E-04	0.2620	1.24E-03
Hydroxylated acylcarnitine	AC-OH	1.28E-03	0.2415	1.34E-02
Deoxyceramide	Cer(m)	2.48E-03	0.2698	2.07E-02
Ubiquinone	Ubiquinone	3.81E-03	0.2465	2.79E-02
Diacylglycerol	DG	4.09E-03	0.2481	2.85E-02
Dihydroceramide	dhCer	5.01E-03	0.2331	3.17E-02
Phosphatidylinositol	PI	6.33E-03	0.2349	3.59E-02
Phosphatidylglycerol	PG	8.38E-03	0.2232	4.39E-02
**Lipid species**				
**Lipid species name**	**Lipid class**	***p*** **-value**	**Beta (SDU)**	**FDR**
Cer(m18:1/18:0)	Cer(m)	1.84E-07	0.4000	1.63E-04
Cer(m18:1/20:0)	Cer(m)	2.23E-05	0.3463	4.44E-03
TG(50:1)[NL-16:0]	TG	2.20E-05	0.3450	4.44E-03
TG(50:1)[NL-18:1]	TG	3.37E-05	0.3405	4.44E-03
TG(50:2)[NL-18:2]	TG	3.18E-05	0.3393	4.44E-03
TG(49:1)[NL-17:1]	TG	2.89E-05	0.3366	4.44E-03
TG(51:2)[NL-17:1]	TG	5.23E-05	0.3354	5.17E-03
TG(54:2)[NL-18:0	TG	6.36E-05	0.3205	5.17E-03
TG(54:6)[NL-20:4]	TG	5.45E-05	0.3069	5.17E-03
TG(53:2)[NL-17:1]	TG	8.04E-05	0.3232	5.76E-03
PI(16:0/16:1)	PI	8.46E-05	0.2901	5.76E-03
TG(51:2)[NL-17:0]	TG	1.13E-04	0.3252	6.22E-03
TG(54:5)[NL-20:4]	TG	1.12E-04	0.2972	6.22E-03
PI(16:0/20:4)	PI	1.08E-04	0.2935	6.22E-03
TG(50:2)[NL-16:1]	TG	1.54E-04	0.3199	6.66E-03
TG(50:3)[NL-16:1]	TG	1.65E-04	0.3182	6.66E-03
TG(52:1)[NL-18:1]	TG	1.64E-04	0.3117	6.66E-03
TG(48:1)[NL-16:1]	TG	1.84E-04	0.3057	6.66E-03
TG(52:2)[NL-18:2]	TG	1.88E-04	0.3028	6.66E-03
TG(50:6)[NL-20:4]	TG	1.40E-04	0.2840	6.66E-03

*SDU, standard deviation units*.

**Figure 2:**
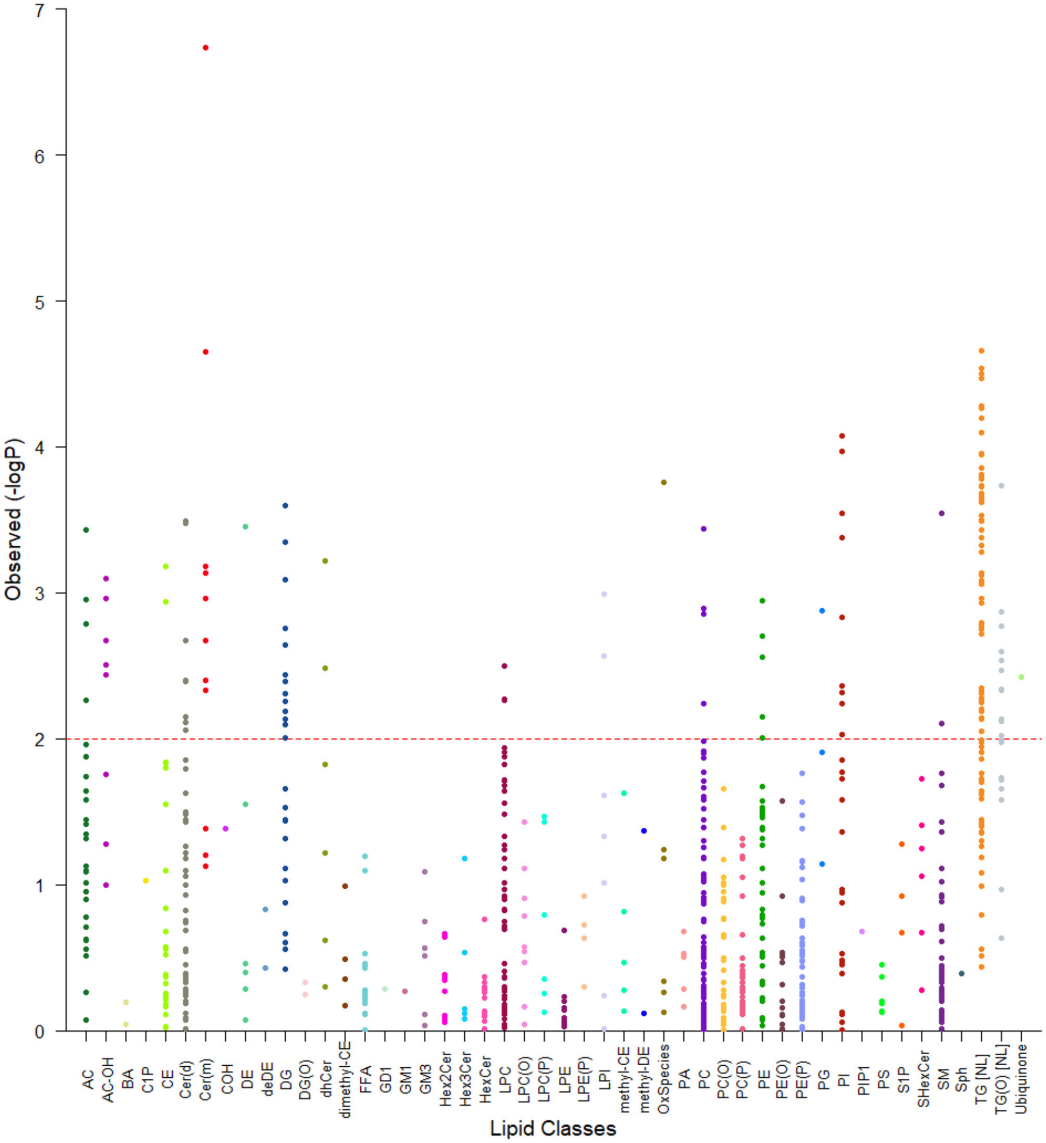
Tornado plot of plasma lipidome associations with EAT volume. The figure shows all lipid species within their specific classes and the degree of association with EAT volume (FDR). Each dot represents a lipid species and each color a lipid class. The dotted line represents an FDR of 0.05. All species above the dotted line were considered as significantly associated with epicardial fat volume.

**Figure 3:**
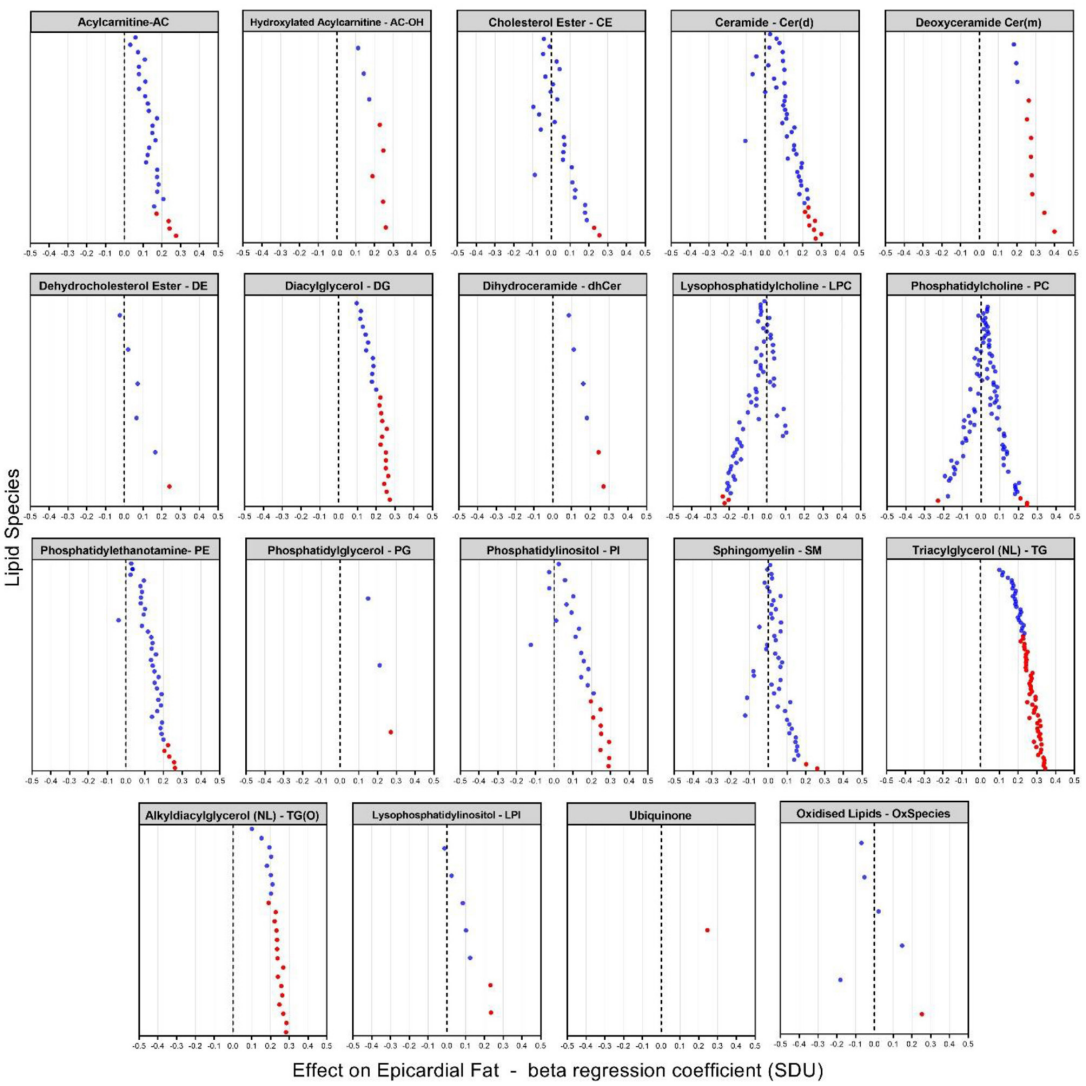
Plasma lipidome associations with EAT volume [beta coefficient of regression (SDU)]. The figure shows significant increases and decreases in lipid levels in 19 classes of lipids with species having an FDR < 0.05 (indicated by the red dots). Each dot represents a lipid species within the specific lipid class.

We next examined the association of lipid species with EAT volume using SOLAR, analyzing 799 lipid species from the 48 classes. After appropriate adjustments described in methods, our plasma lipidome analyses showed that 135 lipid species belonging to 19 lipid classes were significantly associated with EAT volume with an FDR <0.05 as shown in Figures 2, 3. The list of lipid species with their respective average concentration, concentration range and number of lipid species within the class with an FDR < 0.05 are shown in Table 2. The full list of lipid classes and species with *p*-value, beta coefficient of regression (SDU) and FDR analysis are shown in Supplementary Tables S2, S3, respectively.

After appropriate adjustment for the effective number of tests in our plasma lipidome analyses in SOLAR, the EAT volume was associated with the increase of 131 lipids and decrease of 4 individual lipid species. The strongest associations observed were with two deoxyceramide species Cer(m18:1/18:0) and Cer(m18:1/20:0) with beta coefficient of regression of 0.40 and 0.34 and *p* = 1.63 × 10^−4^ and *p* = 4.44 × 10^−3^, respectively. This association demonstrated, in our study, that a larger EAT volume was correlated with an increased plasma concentration of deoxyceramide. The 20 species of lipids with the most significant *p*-values are described in Table 3 and shown in Figure 3. Interestingly, these lipid species belong to only 3 classes of lipids: deoxyceramide, triacylglycerol and phosphatidylinositol. In addition, only species from an extra 6 classes of lipid showed significant associations (Table 3). These additional species belong to the classes: hydroxylated acylcarnitine, alkyldiacylglycerol, ubiquinone, diacylglycerol, dihydroceramide, and phosphatidylglycerol. Additionally, we observed substantial heterogeneity of effects within 10 lipid classes (cholesterol ester (CE), ceramide (Cer(d)), deoxyceramide (Cer(m)), dehydrodesmosterol ester (deDE), lysophosphatidylcholine (LPC), phosphatidylcholine (PC), phosphatidylethanolamine (PE), phosphatidylinositol, sphingomyelin (SM) and lysophosphatidylinositol (LPI). Interestingly, one class (PC) showed both significant increasing (three species) and decreasing (one species) associations with EAT volume, suggesting the potential for dynamic feedback within class, most likely driven by the fatty acids.

The associations observed with the plasma lipidome measures were more significant than those observed for standard clinical lipid markers. We examined the associations of the clinical lipid measures and EAT in this sample and found the associations to be *p* = 0.01 for total cholesterol, *p* = 0.008 for calculated LDL, *p* = 0.0008 for HDL and *p* = 4.32 × 10^−6^ for triglyceride levels. Triglyceride levels were the only clinical lipid measure nearing the level of significance observed in the lipidome measures, particularly for the triacylglycerol (TG) species. This is to be expected as the triglyceride measure is the sum composition of all triacylglycerol species measured in our analysis. In contrast the many other species of ceramide, deoxyceramide and phospholipid species containing polyunsaturated fatty acids are not strongly correlated with clinical lipid measures and so are providing independent information about EAT. For standardized phenotypes, *p*-values are directly related to observed effect sizes (e.g., residual correlation). Therefore, the improved *p*-values directly relate to larger associations and hence greater biological significance.

## Discussion

Given that CVD is the leading cause of death in the US and much of the world, the identification of novel risk factors is essential to help alleviate the substantial health and economic burden. So, novel insights into biological mechanisms that predispose individuals to CVD hold the promise of a significant reduction in this considerable economic burden. It is well-known that a relationship exists between lipid variation and CVD, but the mechanisms are unclear. Modern genomic technologies can be exploited to rapidly identify genes involved in disease susceptibility. However, the cost-effectiveness of such exploratory endeavors can be greatly augmented if genetic basis to a phenotype is strongly suspected. A recent article by Blanco-Gomez et al. (48) explains nicely the complex interplay between phenotypes and disease at the global level where most major diseases are considered complex phenotypes and are the consequence of intermediate phenotypes causally related to or involved in their pathogenesis. These intermediate phenotypes can go all the way down to the tissue, transcript, and ultimately to genomic level (48). Therefore, intermediate phenotypes such as those derived by CMRI, and lipid profiling as described in this study could lie closer to the genomic level and ultimately the causal action of genes. So, the identification of novel lipid-related endophenotypes that are genetically correlated with CVD offers the potential to discover biomarkers which will quickly lead us to the identification of novel CVD treatments and ultimately preventatives.

The most common risk factors for CVD are deleterious lipid concentrations, obesity, hypertension, and smoking. Additionally, significant health disparities exist in CVD in some minority groups, in both health outcomes and quality of care. Hispanic populations have a significantly worse CVD risk profile, however the prevalence of some CVDs, such as coronary heart disease, are lower than in other ethnic groups, a phenomenon known as the Hispanic Paradox (49). Hispanic individuals face significant socioeconomic challenges, and they tend to have low rates of disease awareness and health insurance and are less likely to seek treatment. A recent American Heart Association statement on the health burden of CVD in Hispanics acknowledges insufficient understanding of Hispanic heart health and supports the need for more Hispanic CVD research (49, 50). The work of our group in Mexican American individuals, has demonstrated a role for these lipid species and the measurement of CMRI-derived phenotypes in CVD disease risk. Our results provide information that lipid measures (which are significantly associated with common complex diseases) and the use of the extended pedigrees of Mexican Americans, along with our novel lipidomic approach and CMRI-derived measures are extremely powerful and cost-effective tools for the identification of the relationship between lipid metabolism and CVD risk.

Heritability of coronary heart disease is estimated to be up to 60% and comes from a variety of family and twin studies (51). CMRI phenotypes, including measures of left ventricle (LV) mass, volume and function, demonstrate high heritability (LV mass heritability ranges from 15 to 84%) (52). In our study, the heritability of epicardial fat volume was 86.29% (*p-value* = 1.8 × 10^−6^ ± 0.1617). This is a validation that EAT volume is significantly genetically regulated and demonstrates the power for identification of intermediate phenotypes in CVD prevention. This study focused on cardiac imaging and lipidomic profile with the ultimate aim of identifying possible lipid phenotypes influencing CVD risk. Focusing on these intermediate phenotypes may direct us more rapidly to CVD risk gene identification.

The fundamental mechanisms potentially responsible for cardiovascular events in overweight individuals remains uncertain. Thus, it is important to ask the question whether increases in epicardial fat, as a component of obesity, may provide one of the links between obesity, coronary atherosclerosis and consequently CVD. Fat in the heart resides in three distinct depots: epicardial, pericardial and intracellular fat. Epicardial fat is located on the surface of the heart especially around the epicardial coronary vessels. It can extend into the heart and intersperse within myocardial muscle fibers (35). There is a significant direct relationship between the amount of epicardial fat and general body adiposity (visceral adiposity) as demonstrated in clinical imaging studies by Rabkin (27, 35). Several lines of evidence support a role for epicardial fat in the pathogenesis of coronary artery disease: first, due to the close anatomic relationship between epicardial fat and coronary arteries (35, 53); second, due to the positive correlation between the amount of epicardial fat and the presence of coronary atherosclerosis (54, 55) and third, due to the ability of adipose tissue to secrete hormones and cytokines that modulate coronary artery atherothrombosis (31). Thus, epicardial fat may be an important factor responsible for cardiovascular disease in obesity (25, 33).

Genetic and epidemiological studies have greatly improved our understanding of pathophysiology underlying the complex of CVDs and have identified several risk factors. Amongst the well-recognized predisposing factors, lipid metabolism plays a central role in the development of CVDs (56, 57). Since the landmark publications from the Framingham study (58), plasma lipids have been recognized as important predictors of future CVD events (59). To assess these events, plasma lipids are routinely monitored by profiling total cholesterol, triglycerides, high-density lipoprotein cholesterol (HDL-C) and low-density lipoprotein cholesterol (LDL-C) (referred as “traditional lipids”). Efforts to understand the role of lipids in CVD pathophysiology had largely focused on these traditional lipids, and to some extent on free fatty acids and lipoproteins. However, human plasma consists of thousands of functionally and chemically diverse molecular lipid species (41, 60). Nevertheless, tremendous advancements in the field of lipidomics has facilitated these efforts to unravel the metabolic dysregulation in complex lipid-related disorders, particularly in CVDs and for the identification of predictive biomarkers beyond traditional lipids (61, 62). Lipids are of central importance for the bioenergetic metabolism of the heart and in the healthy heart, fatty acids (FAs) account for 60–90% of ATP production while glucose provides the remainder (63, 64). When damaged, the heart shifts away from lipids toward a greater reliance on glycolysis, ketone body oxidation, amino acids, and lactate as sources of energy (63, 65). This shift in cardiac metabolism and identification of the metabolic pathways involved remains a challenge and consequently so too does the translation of these metabolic findings to the clinical setting for improved or novel diagnostic/prognostic biomarkers (17). That interdependence of metabolites results in a disease signature which can be used to more precisely identify or predict disease states, which represents the closest link to the phenotype. The biologically simpler nature of these individual lipid species suggest that they may reside closer to the causal action of genes, making them valuable endophenotypes (18). Even though changes in the levels of numerous metabolites have been shown to occur in the failing heart (BCAA, lactate, ketones), specific lipid metabolites appear to consistently change in metabolomic profiles of CVD patients, namely sphingolipids, phospholipids, glycolipids, cholesterol esters, fatty acids and acylcarnitines (63).

Epidemiological and lipidomic studies has shown that specific lipoproteins and their constituent lipids are important factors in the development of metabolic related disorders (9, 10, 66) and CVD specifically (67–69). Glycerolipids are a large group of lipids accounting for a good proportion of total lipids in plasma. Triacylglycerol (TG) is the most abundant lipid class and comprises the bulk of storage fat in tissues. Monoacylglycerol (MG) and diacylglycerol (DG) represent intermediates in the biosynthesis and hydrolysis of TG and function as second messengers in signal transduction processes (70, 71). It has previously been shown that the breakdown and synthesis of triglycerides by DG and MG have a causal effect on CVD risk (13). Sphingolipids are wide range of complex lipids and constitute several hundreds of different species, including ceramides, which function as a precursor for complex sphingolipids (72, 73). Sphingolipids and their precursors, may be involved in the pathogenesis of CVD through multiple pathways including inflammation (74), atherosclerosis (75), and apoptosis (76). Kulkarni et al. have demonstrated a role for diacylglycerols, with four species (each containing palmitic acid) showing significant genetic correlations with hypertension (9). Additionally, Mamtani et al. showed consistent association of ceramides (Cer(d)) with waist circumference where levels were significantly higher in diabetics compared to non-diabetics (*p* = 2.5 × 10^−7^) (10). Alshehry et al. showed that monohexosylceramide (HexCer), dihexosylceramide (Hex2Cer) and lysoalkylphosphatidylcholine (LPC(O)) were associated with cardiovascular events and HexCer and Hex2Cer with cardiovascular death (68). Alshehry et al. found that five species of lipids containing polyunsaturated fatty acids (PUFAs), including phospatidylcholine (PC,) alkenylphosphatidylcholine (PC(P)) and triacylglycerol were inversely associated with CVD risk. In our results, we show that two of these lipids (PC and TG) are associated with epicardial fat volume.

It is now well-established that ceramide metabolism is altered in the context of type 2 diabetes (T2D) and obesity which are both linked to CVD (77, 78). In fact, higher plasma ceramide levels have been associated with visceral obesity, non-alcoholic fatty liver disease, and T2D, which are also predictors of CVDs (79–84). There is an interplay between the level of ceramides in the plasma/tissues and disease development. Hepatocellular ceramides stimulate fatty acid uptake (85) and decrease glucose uptake (85, 86), which enables hepatocytes to favor fatty acids over glucose as a preferred energy source and leads to NAFLD (85). In our study, eight of the 47 ceramide lipids were significantly associated with EAT volume. The most significant species within the Cer(d) class with both FDR value of 6.97x 10^−3^ was Cer(d18:1/18:0) and Cer(d19:1/18:0) (*p*-value 3.31 × 10^−4^). Several studies have found these species to be associated with CVD (82–84) and that ratios of long chain ceramide species Cer(d18:1/16:0) and Cer(d18:1/18:0) were more strongly correlated with negative cardiovascular outcomes than ratios of very long chains of ceramide species Cer(d18:1/24:0) (84). The Cer(d) species associated with EAT volume in our cohort were only long chain ceramide species. Laaksonen et al. (84) found that the ratios of Cer(d18:1/24:0)/Cer(d18:1/16:0) and Cer(d18:1/24:0)/Cer(d18:1/16:0) negatively correlate with coronary heart disease and mortality, and Anroedh et al. found Cer(d18:1/20:0) to be associated with CVD event and death (87). This last lipid species was positively associated with EAT volume in our cohort with (SDU 0.3463; FDR 4.44 × 10^−3^; *p*-value 2.23 × 10^−5^). Another more recent study found that serum sphingolipids are markers of coronary artery disease, independent of cholesterol (88), further solidifying the strong correlation between these species and negative cardiac outcomes. These dual (positive or negative) ceramide actions may be due to manipulations of the *de novo* ceramide synthesis pathway suggesting that certain ceramide species are deleterious, whereas others are beneficial (77, 89–91). Those containing the C16 or C18 acyl chain (77, 89, 90) and a double bond (i.e., ceramides, not dihydroceramides) (92) in the sphingolipid backbone are particularly harmful. Thus, plasma ceramide levels can be used as clinical biomarkers to predict risk of cardiovascular death. Another lipid class that we found to be significantly associated with EAT volume was dihydroceramide (dhCer). This lipid is the precursor of ceramide in the *de novo* synthesis pathway. Ceramides and their derivates are involved in many, if not all, essential cellular process such cell growth, cell adhesion and migration, senescence, apoptosis, inflammation, immune system and angiogenesis (73, 93). Ceramide synthesis and accumulation are influenced by multiple factors, which include excessive supply of substrates, systemic inflammation, oxidative stress and the microbiome (94). Therefore, the many roles played by ceramides and derivates in cardiometabolic health and diseases are associated with this connection between excessive supply of lipids and inflammation (95). The ceramide family of species has been reported to have a strong correlation with CVD risk in the literature. We found that 2 of 6 measured dhCer species (dhCer(d18:0/24:1) and dhCer(d18:0/22:0)) were significantly associated with EAT volume. Other authors have reported the existence of a strong correlation between CVD and increased dihydroceramide levels. Elevated levels of dihydroceramides have been found in atherosclerotic plaques (75). The role this increase in dihydroceramides plays in plaque stability is still debatable, since the extracellular addition of dihydroceramides to human aortic smooth muscle cells did not cause apoptosis, whereas addition of ceramides did (75). One atypical sphingolipid (deoxyceramide -Cer(m)) was also associated with EAT in our study, with the two most significant FDR results. These ceramides are neurotoxic sphingolipids which are formed by the enzyme serine palmitoyltransferase (SPT). Pathologically elevated Cer(m) levels were found in patients with MetS or T2D (96–99). The mechanisms which underlie this increased Cer(m) formation in metabolic disorders is not understood. Low but detectable Cer(m) levels are generally found in plasma - also of healthy individuals. However, patients with MetS or T2D have significantly elevated plasma Cer(m) levels (98, 99). These observations may reflect an underlying interaction between the severity or control of T2D in this population and cardiovascular risk.

The glycerophospholipids, also known as phospholipids, are the major structural component of cell membranes and are involved in various biological processes including inflammation (72). It has been shown that there is a marked difference in the lipidome of adipose tissues depending on their epicardial or subcutaneous origin (SAT) (21). Barchuk et al. found a specific enrichment in sphingolipids, phosphatidylcholine (PC), phosphatidylethanolamine (PE), and phosphatidylethanolamine plasmalogens (PE(P)) in EAT compared with SAT. They observed that EAT was rich in PC and in PE. Our results corroborate this finding, identifying a total of 127 different species of PC and PE, accounting for almost 16% of total lipid profile. From those, a total of nine lipids (4 PC and 5 PE) were associated with EAT in our cohort. Barchuk et al. (21) also observed a specific lipidomic signature of EAT in coronary artery disease (CAD) patients, identifying 97 lipid species that discriminated patients with and without CAD. They also observed that patients with CAD exhibited more ceramides, diacylglycerides, monoacylglycerides, and less unsaturated triacylglycerols in their EAT. This group also found that that the EAT lipidome was independent to that of SAT, possibly indicating a specific high metabolic activity, or a beige associated phenotype. Although we did not evaluate cardiovascular disease between the individuals with high EAT in our cohort, the lipid profile associated with EAT was basically the same as found by Barchuk et al., where the majority of the lipids associated with EAT were from sphingolipids (ceramides, dihydroceramides, 1-deoxy-sphingolipds, sphingomyelins) and glycerolipids (DG and TG) (21). Our results suggest that saturated fatty acids (FAs) on the TG species show strong association with EAT. The majority of the FAs on the top 28 TG species significantly associated with EAT were saturated. Stegemann et al. in a population-based study, identified a specific cluster of triacylglycerol species with saturated and monounsaturated acyl chains as most consistently associated with CVD (100). The association between saturated/monounsaturated fatty acids and cardiovascular risk was also evident in or study. Recently, Tomasova et al., found a higher content of phosphatidylcholine and decreased level of phosphatidylethanolamine (18:0/20:4) in EAT compared with SAT (101). Although our study didn't measure SAT volume for comparison with EAT, we did find high numbers of PCs and PEs associated with EAT volume, where high concentrations of these lipids was observed in individuals with high epicardial volume. These observations may reflect an underlying interaction between the severity or control of T2D and CVD risk.

These associations suggest that there are multiple factors that influence lipid homeostasis and presumably CVD risk. The effects of these lipid species on epicardial fat volume, after adjustment for established risk factors, likely reflects both environmental and genetic factors not currently considered in CVD risk.

## Limitations

Some limitations of the present study need to be considered. First, our findings should be seen as indicative and need confirmation by replication in independent cohorts. All study participants were Mexican Americans, so, it is not possible to generalize these results to other ethnic groups. Future studies need to investigate the similarities and differences of lipid associations and CVD risk in different ethnic populations. Second is the limitation of all lipidomic studies where the coverage is incomplete. In this study, we used a targeted approach that has enabled us to measure 799 lipid species from 48 different lipid classes, providing a broad, but still incomplete, coverage of the lipidome. Third, the high variance associated with lipidomic measurements can reduce the strength of observed associations. Lastly, all inferences in this study are based on cross-sectional data. The associations therefore do not automatically imply a causal role of plasma lipid species in the pathogenesis of CVD. The main goal of the study was to query the existence of potential correlations in EAT volume and specific lipid species for prediction of CVD risk.

## Conclusion

In conclusion, using a novel integrative approach by combining plasma lipidomics and CMR imaging of epicardial fat volume, we have shown that specific lipid abnormalities correlate with cardiovascular disease risk. Our findings should be considered as preliminary and the possible role of these triacylglycerols and/or ceramides in the development or prevention of CVD warrant further investigations. On a general level, our study also provides a framework for linking plasma lipidomic markers not only with clinical endpoints, but also with the more subtle intermediate phenotypes, as derived from medical imaging of potential pathophysiological relevance.

## Data Availability Statement

The original contributions presented in the study are included in the article/Supplementary Material, further inquiries can be directed to the corresponding author/s.

## Ethics Statement

The studies involving human participants were reviewed and approved by both the Institutional Review Board at University of Texas Health Science Center San Antonio and the Institutional Review Board at University of Texas Rio Grande Valley. The patients/participants provided their written informed consent to participate in this study.

## Author Contributions

LM, JB, and JC contributed to the conception and design of the study. AL organized the study data, performed descriptive statistics, generated tables and figures, and wrote the first draft of the manuscript. RD was responsible for the recruitment of the SAFHS participants in the study. GC performed the cardiac MRI acquisition and calculation of EAT volume. KH, CG, and TD performed lipidomic profiling and quantification of lipid species and classes. PM was responsible for overseeing the lipidomic profiling, interpretation of the data, and wrote sections of the manuscript. JB directs the SAFHS cohort and was responsible for overseeing the statistical analysis and wrote sections of the manuscript. MA, MK, and VD were responsible for the statistical analyses in SOLAR and for generating some of the figures. JC directed the study and wrote sections of the manuscript. All authors contributed to manuscript revision, read, and approved the submitted version.

## Funding

This work was supported in part by National Institutes of Health (NIH) grants P01 HL045522 (SAFHS data collection), R01 HL140681 (lipid profiling), R37 MH059490 (analytical methods and software used), R01 EB015611 (analytical methods and software used), Eli Lilly and Company (cardiac imaging) and a grant from the Valley Baptist Legacy Foundation for Project THRIVE (biorepository). This work was conducted in part in facilities constructed under the support of NIH grant C06 RR020547. PM was supported by a L3 Investigator grant from the National Health and Medical Research Council of Australia (2009965). KH was supported by a National Health and Medical Research Council of Australia investigator grant (1197190). The funders were not involved in the study design, collection, analysis, interpretation of data, the writing of this article or the decision to submit it for publication.

## Conflict of Interest

LM was employed by Eli Lilly and Company. The remaining authors declare that the research was conducted in the absence of any commercial or financial relationships that could be construed as a potential conflict of interest.

## Publisher's Note

All claims expressed in this article are solely those of the authors and do not necessarily represent those of their affiliated organizations, or those of the publisher, the editors and the reviewers. Any product that may be evaluated in this article, or claim that may be made by its manufacturer, is not guaranteed or endorsed by the publisher.
